# A State-of-the-Art Roadmap for Biomarker-Driven Drug Development in the Era of Personalized Therapies

**DOI:** 10.3390/jpm12050669

**Published:** 2022-04-21

**Authors:** Victoria Serelli-Lee, Kazumi Ito, Akira Koibuchi, Takahiko Tanigawa, Takayo Ueno, Nobuko Matsushima, Yasuhiko Imai

**Affiliations:** 1 Clinical Evaluation Sub-Committee, Medicinal Evaluation Committee, Japan Pharmaceuticals Manufacturers Association, 2-3-11, Nihonbashi Honcho, Chuo-ku, Tokyo 103-0023, Japan; akira.koibuchi@astellas.com (A.K.); takahiko.tanigawa@bayer.com (T.T.); takayo.ueno@bms.com (T.U.); nmatsus3@its.jnj.com (N.M.); 2 Eli Lilly Japan K.K., 5-1-28 Isogamidori, Chuo-ku, Kobe 651-0086, Japan; 3 Daiichi Sankyo Co., Ltd., 1-2-58 Hiromachi, Shinagawa-ku, Tokyo 140-8710, Japan; ito.kazumi.fr@daiichisankyo.co.jp; 4 Astellas Pharma Inc., 2-5-1 Nihonbashi-Honcho, Chuo-ku, Tokyo 103-8411, Japan; 5 Bayer Yakuhin Ltd., 2-4-9, Umeda, Kita-ku, Osaka 530-0001, Japan; 6 Bristol Myers Squibb K.K., 6-5-1 Nishi-Shinjuku, Shinjuku-ku, Tokyo 163-1334, Japan; 7 Janssen Pharmaceutical K.K., 3-5-2, Nishikanda, Chiyoda-ku, Tokyo 101-0065, Japan

**Keywords:** biomarker-driven drug development, real-world data/evidence, personalized medicine, genome medicine, data ecosystem, ecosystem for personalized therapies, disease blueprint, clinical trial renovation, Japan

## Abstract

Advances in biotechnology have enabled us to assay human tissue and cells to a depth and resolution that was never possible before, redefining what we know as the “biomarker”, and how we define a “disease”. This comes along with the shift of focus from a “one-drug-fits-all” to a “personalized approach”, placing the drug development industry in a highly dynamic landscape, having to navigate such disruptive trends. In response to this, innovative clinical trial designs have been key in realizing biomarker-driven drug development. Regulatory approvals of cancer genome sequencing panels and associated targeted therapies has brought personalized medicines to the clinic. Increasing availability of sophisticated biotechnologies such as next-generation sequencing (NGS) has also led to a massive outflux of real-world genomic data. This review summarizes the current state of biomarker-driven drug development and highlights examples showing the utility and importance of the application of real-world data in the process. We also propose that all stakeholders in drug development should (1) be conscious of and efficiently utilize real-world evidence and (2) re-vamp the way the industry approaches drug development in this era of personalized medicines.

## 1. Introduction

The aim in drug development is to validate a clinically efficacious dose of a new drug for a defined disease state, i.e., finding the dose that will be effective for a defined group of patients while causing the least side effects. In drug development today, where a large proportion of new drug entities are targeted agents with specific molecular targets, the interpretation of this aim should be re-defined and the approach to drug development transformed. There are two parts to this transformation:

“*Clinically efficacious dose*”—it is well established that there are individual differences that lead to differences in clinical efficacy and severity of side effects caused by drugs. In oncology, clinical biomarkers are used to identify individuals who may respond better to targeted therapies. Pharmacogenomic (PGx) markers are also widely used now to determine dosages or identify patients who may have an adverse side effect to a drug.

“*Defined disease state*”—in May 2017, the Food and Drug Administration (FDA) approved pembrolizumab for microsatellite instability-high (MSI-H) or mismatch repair deficient (dMMR) solid tumors—the first regulatory approval of an indication defined by biomarkers [1]. This signified the beginning of biomarker-based diagnoses in the clinic.

The biomarker is at the core of the two concepts. Identifying clinically significant biomarkers and ensuring that there are robust methods of evaluating it is central to patient safety and delivering clinical efficacy.

Biomarker-driven drug development embeds our knowledge of disease etiology into clinical trial designs with the aim of de-risking the process and improving success rates [2,3,4]. Over the past two decades, with advances in biotechnology, drug development and healthcare has greatly advanced especially in the field of genomic and precision medicine. Specifically, in recent years comprehensive genome profiling (CGP) using next-generation sequencing (NGS) has provided the ability to molecularly profile cancers. This provides information on the complexity of the disease and potentially identifies actionable mutations to which available targeted therapies can be prescribed. Nevertheless, clinical efficacy percentages even within the biomarker-selected population are sub-optimal. This suggests the heterogenous nature of disease manifestations in different individuals, the details of which are still unknown.

Using an array of next-generation technologies, biomarker assays today can generate high-resolution data that provide biological information down to the single-cell level [5,6,7,8]. Accumulating omics data, clinical data, electronic health records and wearable device data are contributing to the “data overload” [9] in healthcare. The ability to apply analytics and artificial intelligence (AI) to mine this real-world data (RWD) for insights is accelerating the progress of our understanding of various disease etiologies and drug interactions [10], and is changing the way we approach science and drug development.

## 2. Background

**Figure 1:**
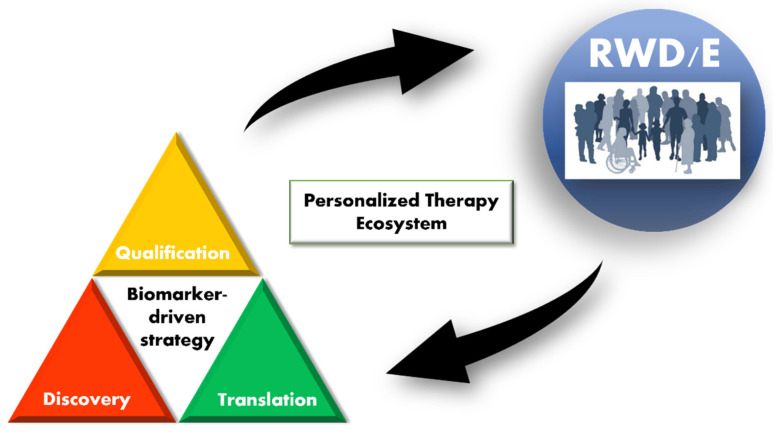
Personalized therapy ecosystem (PTE). A pictorial representation of a PTE. A biomarker “lifecycle” is broken down into 3 stages—discovery, translation and qualification. Biomarker testing in the clinic generates real-world data/evidence (RWD/E) that feeds back into (supports) all stages of biomarker-driven drug development. Increased efficiency in development leads to more clinically validated biomarkers that can be used in clinical practice.

Amidst this dynamic landscape, the Japan Pharmaceuticals Manufacturers Association (JPMA) had put together a working group to address the topic of “Clinical Biomarkers in Personalized Therapies”. The aim of the working group was to increase awareness and advocate for transformation in this field. The group comprised representatives from pharmaceutical organizations. We summarized the current state of biomarker-driven drug development as a “Personalized therapy ecosystem” (PTE) and represented this pictorially (Figure 1). The PTE was conceived based on examples of the use of RWD/evidence (RWD/E) in the process of drug development, which we review in the sections below. Since the PTE comprises multiple stakeholders involved in biomarker and drug development, the goal of this review is to engage with all stakeholders to instill a common understanding.

Our working group had also conducted a survey (via a questionnaire sent to participating member companies of the JPMA) on the biopharmaceutical industry in Japan in 2020. The survey assessed the current use of biomarkers and the application of RWD in drug development. Full results were shared at the 42nd Annual Scientific Meeting of the Japanese Society of Clinical Pharmacology and Therapeutics [11]. The survey revealed key issues and challenges faced by drug makers in Japan, which are the basis of our proposals.

In this review, the lifecycle of a biomarker is broken down into three stages—discovery, translation and qualification. In each of these stages, we briefly review current practices that support our idea of a PTE.

By the juxtaposition of traditional/current drug development processes with latest breakthrough concepts in the field, we propose a new “state-of-the-art” roadmap for drug development, in the era of personalized therapies.

## 3. Definitions and Scope

“Clinical biomarkers”, as defined in the BEST (Biomarkers, EndpointS and other Tools) [12] resource, put together by the FDA-National Institutes of Health (NIH) Biomarker working group, is “ a defined characteristic that is measured as an indicator of normal biological processes, pathogenic processes, or responses to an exposure or intervention, including therapeutic interventions. Molecular, histologic, radiographic, or physiologic characteristics are types of biomarkers. A biomarker is not an assessment of how an individual feels, functions, or survives”. In our proposed PTE, we use this broad definition and use the term “biomarker” to refer to all quanta of measurements—from single molecular targets to whole genome sequence, to multiplex biomarker algorithms, to omics-signatures, to high resolution medical images and digital biomarkers.

We refer to a wholistic biomarker assessment of an individual’s disease state as a *disease blueprint*, which we define as the true omni-level etiology of an individual’s disease state. Like an architectural blueprint provides all the details required to construct a building, a disease blueprint reveals the pathophysiology of a disease. Depending on the disease, this blueprint could be derived via the integration of various types of biomarker information—next-generation mutli-omic assay data, medical images and even ambulatory vital sign measurements. With this blueprint at hand, targeted therapies can be accurately prescribed to patients. In the management of chronic diseases, the blueprint may also guide treatment strategies, ensuring optimization.

The concept of “personalized approach” or “right drug to right patient” takes on several nomenclatures in the literature—“targeted therapy”, “precision medicine” and “personalized medicine”. In this review, we utilize various nomenclatures depending on the context. In our proposed PTE, the term “personalized therapy” refers to a therapeutic method or drug prescribed to a patient based on their disease blueprint.

An “ecosystem” typically refers to an interconnected network. In biology, it is a network of interdependent living organisms. In our proposed PTE, all stakeholders in drug development—drug makers, in vitro diagnostic (IVD) makers, biotechnology industry, healthcare industry and supporting infrastructure, regulatory agencies, academia, patients and advocacy groups—are participating members. All members are interdependent for the unified benefit of eventually delivering personalized therapies to patients.

The scope of our review encompasses various aspects of biomarker-driven drug development for all relevant stakeholders to understand and consider. Since cancer is the most mature therapeutic area practicing targeted therapies, most of our cited examples are oncology related. However, we do not limit our recommendations and discussion to oncology.

## 4. Discovery

Biomarker discovery ties in closely with new drug discovery. Identifying biologically relevant and druggable targets for diseases is a key challenge in drug development. Evidence shows that deep understanding of disease pathophysiology is an important factor of success [13,14,15]. There are limitations to extrapolating efficacy, ADME (absorption, distribution, metabolism and excretion) and toxicity profiles of drugs in humans from nonclinical studies using traditional phenotypic animal disease models and/or immortalized cell lines. This section summarizes current RWD/E-driven approaches taken in this area. A summary of the approaches is presented in Table 1.

### 4.1. Big Data and Computational Approaches

Large-scale, nationwide initiatives to sequence the genomes of large populations provide a valuable data source to mine for research and target identification. Examples include government-led initiatives such as the 100,000 Genomes Project (UK) [16] and the million-genome Precision Medicine Initiative (US). In Japan, the Tohoku Medical Megabank (ToMMo) Project is the largest cohort, comprising clinical, genomic and multi-omics data from more than 150,000 participants [17].

Genomic analyses such as genome-wide association studies (GWAS) are traditionally conducted on large cohort data to identify quantitative trait loci in specific populations, which may shed insight into disease mechanisms. More recently, GWAS is further combined with functional genomic strategies to shed further insight into disease pathology [18]. Disease-specific molecular profiles have been identified in publicly available databases, and such insights are used for discovery, repurposing and development [19,20].

Quantitative systems pharmacology (QSP) is a powerful method whose use signifies a paradigm shift from a single gene to a multi-modal approach. QSP integrates pharmacokinetic and pharmacodynamic data with the “system” being studied. It also provides a quantitative framework for the integration of diverse omics data sources and translation of molecular data to clinical outcomes. Multi-omics data are increasingly being used to deepen our understanding of the pathophysiology of diseases and are suggested to be novel tools for discovery of drug targets and disease-associated biomarkers [21,22,23].

Utilizing omics analyses and databases, biological networks can be mathematically modeled, enabling quantitative analyses of normal biological regulation versus dysfunction in pathophysiological states. Multi-scaled QSP-network models (i.e., mathematical models based on multiple temporal and spatial scales—molecules, cells, tissues, organs, organisms, and patients) of networks involved in disease progression can be potentially used to define a wholistic profile of a disease—or a *disease blueprint*. Well-established models could potentially also provide the ability to predict the effects of a drug candidate on pathophysiology [24,25], thus aiding in the optimization process. Most recently, the mapping of the human interactome (genome-proteome interactions) has reached an extensive level of 53,000 high-quality protein-protein interactions [26]. The application of network science on such models can transform the way biomarkers are discovered and even the way diseases are defined [27].

Curated databases such as The Cancer Genome Atlas (TCGA) are an invaluable source of knowledge and is becoming a key tool in biomarker discovery. It archives genomic, transcriptomic, epigenetic and proteomic information collected from over 20,000 primary cancers and matched normal samples for 33 cancer types [28]. On the TCGA website alone, there are 72 listed publications during the period from 2008 through 2021. A crude search on the PubMed Central database in January 2022 revealed 12,512 hits with “TCGA” found in the title or abstract of the article. Further refinement of the search to look for mentions in the “Methods” section revealed 328 hits, showing the value of TCGA data as a tool in research. Other than being a source of RWD that can be mined for new discoveries [29,30,31,32,33], it also acts as a useful reference dataset in clinical trials and biomarker/omics signature validation studies [34,35,36].

In Japan, cancer precision medicine initiatives such as SCRUM-Japan [37] (solid tumors) and MASTER KEY project [38] (rare cancers) are currently ongoing. These function as screening platforms to identify cancer patients with actionable mutations who may be eligible to participate in ongoing clinical trials. The accumulated genomic, transcriptomic, and clinical data collected in these initiatives contribute to a growing clinico-genomic database from which novel biomarkers have now been identified [39]. There is also an effort to establish a Japanese Cancer Genome Atlas (JCGA) using data generated from tumor samples collected from Japanese patients [40]. In this effort, clinical samples from more than 5000 cancer patients were subject to DNA and RNA sequencing and gene expression profiling. Driver and actionable genomic alterations in cancer-related genes were cross-referenced to existing databases and literature to form a curated catalogue of cancer genome information.

Increases in the utilization and mining of such curated data may offer invaluable insights into population-specific, disease outcome-related traits, and these should be leveraged where and when possible.

### 4.2. Experimental Approaches

Application of patient derived cells in in vitro studies and in vivo patient-derived xenograft models is a powerful method for evaluating and optimizing drug candidates [41,42]. Human-induced pluripotent stem cells (hiPSCs) are also useful in the drug target discovery process. hiPSCs, particularly when differentiated into three-dimensional multicellular models such as spheroids or organoids, may be used as models to mimic in vivo pathophysiology and pharmacological responsiveness. CRISPR/Cas9-based gene editing is more specific and powerful than traditional knockout methods in producing loss-of-function phenotypes and is now being employed to create isogenic disease models for drug screening [43] and target identification [44]. By integrating the CRISPR/Cas9 screening output with other lines of data, including real world patient data, a quantitative framework was generated for screening drug candidates [45]. This example demonstrated the value of combining experimental and computational data-driven approaches.

In Japan, clinical samples for research can be obtained via collaboration with academia, from public biobanks, or by prospectively collecting stored specimens in clinical trials. Samples in public biobanks come from hospitals and research institutions, where stakeholders cooperate to collect and manage human tissue, cells and/or biofluid samples along with donors’ background and health data [46,47]. However, processes for sample and information distribution from biobanks in Japan may sometimes be tangled and limitations around personal data and privacy often apply for commercial use. On the contrary, patient samples obtained from clinical trials can be used for research and development (R&D) under appropriately documented informed consents. Here, although the necessary patient information is appropriately collected and managed, sample size may sometimes be limited.

It is therefore important for stored samples from public biobanks and/or clinical trial sample repositories to be realistically available for discovery and translational research.

## 5. Translation

Translational research involves bringing scientific discoveries or basic research concepts into the clinic. In this section we review the processes involved in taking a biomarker from the R&D phase to its use in a clinical setting, including the technical assay validation process. A well-established process is the development of a companion diagnostic (CDx). A large part of this process is the selection and analytical validation of an appropriate assay method to measure the biomarker of interest. Complementary diagnostics, biomarkers of prognostic value, PGx biomarkers or biomarkers used as surrogate measures also require robust assay methods to be available before implementation in clinical studies. The BEST resource 2020 states that validation is important for ensuring that the test, tool or instrument is adequate for its proposed use [12]. Validation of a biomarker in drug development comprises of 2 main pillars:

(1) Analytical validation of the assay methodology to evaluate performance characteristics such as precision, accuracy, specificity, selectivity, sensitivity, analytical range, interference, and sample stability to broadly name a few [48,49]
(2) Clinical validation/qualification to demonstrate the relationship of a biomarker with the clinical outcome it is posited to be associated with.

The spectrum of biomarkers used in drug development spans a wide range of definitions and intended applications (proposed use) frequently also referred to as context of use (COU). The required rigor of analytical validation should correspond to the clinical or regulatory risk associated with the COU of the biomarker. This ensures that the evidence generated by the biomarker assay is sufficiently robust to support the intended application or claim. This concept of “fit-for-purpose” is an iterative approach to biomarker validation. For example, a biomarker assay validated for use in exploratory projects or early phase trials may be basic or of less rigor, but as the role of the biomarker matures during drug development, additional validation requirements may have to be reconsidered as the COU and associated regulatory risks evolve. The intent of this fit-for-purpose concept which was first proposed in 2005 [50], was to provide guidance and increase the efficiency of incorporating biomarkers into drug development. This concept is now widely applied and also described and applied in the FDA draft guidance, the “Biomarker Qualification: Evidentiary Framework” [51].

Two industry white papers published by the Critical Path Institute extensively summarize and provide recommendations for analytical validation of various types of biomarkers based on assay method [48,49].

In a CDx development program, determining the clinically relevant threshold, sometimes known as “cut-point” or “cutoff” is a key requirement in clinical validation. This threshold value should be clinically relevant in the appropriate patient population [52]. Ensuring good technical performance of the biomarker assay at and around the range of the clinically relevant cut-point is crucial to accurately differentiate between the biomarker “positive” and “negative” populations. This process and the qualification of the clinical utility of the biomarker (reviewed in the next section) may be iterative. A well-known case-study of cut-point establishment is in the case of PD-L1 as measured by IHC, as a CDx for pembrolizumab [53].

In many cases, the process of analytical and clinical validation being iterative contributes to the overall development process being labor- and cost-intensive. The FDA, recognizing that there is wealth of real-world clinical lab data that can be potentially utilized as evidence to support the development of IVDs, issued two guidance’s—in August 2017, “Use of RWE to Support Regulatory Decision Making for Medical Devices” [54] and then in April 2018, “Use of Public Human Genetic Variant Databases to Support Clinical Validity for Genetic and Genome-based in vitro diagnostics” [55]. A report was also recently published by the Medical Device Innovation Consortium on using RWE in pre- and post-market regulatory decision making for IVDs [56].

A well-cited example of the use of RWD in supporting clinical performance validation is the MSK-IMPACT cancer panel [57]. The MSK-IMPACT cancer panel, originally a laboratory-developed test, obtained authorization from the FDA under the 510(k) framework in 2017. The source of evidence for clinical performance was from a clinical evidence curation resource—OncoKB [58]. This example highlights synergies that can be achieved when data are thoughtfully curated and appropriately shared—as these data become the invaluable evidence that eventually support the broader use of the biomarker assay in clinics.

## 6. Qualification

The “Qualification” of a biomarker demonstrates the clinical utility of the biomarker. “Clinical utility” as defined by the National Cancer Institute’s (NCI) dictionary of genetic terms is the “likelihood that a test will, by prompting an intervention, result in an improved health outcome”. Depending on the purpose and COU of a biomarker, there are various approaches to validating clinical utility of biomarkers in different settings and disease populations.

Choice of study design is critical in demonstrating clinical relevance and utility. This section reviews designs employed to test various biomarker hypotheses in different contexts.

### 6.1. Mendelian Randomization

Mendelian randomization (MR) is an instrumental variable approach widely used in observational studies to strengthen causal inferences made retrospectively from data. It is analogous to a randomized controlled trial, except that in MR, a germline genetic variant that is known to be strongly associated with an exposure, is utilized as a proxy for the risk factor of interest [59]. Mendelian randomization has been widely used in correlating disease risk factors with biomarkers [60,61] and is used to aid in the identification of potential drug targets or biomarkers. With the accumulation of genomic data in databases, data from GWAS are also improving our understanding of genetic variants associated with diseases. Using this knowledge in MR studies, causal relationships between a disease or clinical outcome with a biomarker or risk factor of interest have been established [62,63].

### 6.2. Single Biomarker-Driven Clinical Trials

Pivotal clinical trials evaluating the use of a single-biomarker CDx with a therapeutic product typically employ an “enrichment design” or a “biomarker-based strategy design”.

“Enrichment designs” enroll only biomarker-positive patients to evaluate the safety and efficacy of the treatment in the biomarker-positive population. This design is evident in the pivotal trial for trastuzumab in HER2-positive breast cancer patients [64]. Although efficient if the biomarker correlates well with the disease etiology, this approach raises the question of whether the investigational drug may also be potentially effective in the biomarker-negative population, since data from this group of subjects are not obtained in the study.

“Biomarker-based strategy design” randomizes subjects to either a biomarker-based strategy arm or a control arm (non-biomarker-based strategy). The biomarker-based strategy arm is further sub-divided into the biomarker-positive group, receiving the investigational drug, while the biomarker-negative group receives standard of care. In the control arm, subjects are assigned to the standard of care treatment. Some trial designs may also include a sub-group within the control arm receiving an investigational drug [65]. In the case of developing a CDx, utilizing this design may generate robust data to support clinical validation of the IVD, but the operational feasibility, scale and cost is high.

Still, several successful examples exist for biomarker-driven designs. Vemurafenib, a tyrosine kinase inhibitor targeting previously untreated melanoma harboring the BRAF-V600E-mutation, was shown to improve rates of overall survival and progression-free survival [66]. Erlotinib and gefitinib are approved for epidermal growth factor receptor mutation-positive bronchial carcinomas which occur in 10% of the Caucasian and 30% of the Asian lung cancer patient population [67,68].

Having an analytically validated biomarker assay early in drug development and successful partnership between drug and IVD makers in the case of a CDx development remains a challenge that is widely discussed to date. We briefly comment on these challenges in our discussion.

While trials selecting patients based on a single biomarker have demonstrated overall benefit in clinical trials, there remains a sub-group of the selected patients who do not experience clinical benefits. The need to assess biomarkers more comprehensively and efficiently has presented drug trial sponsors with the challenge of looking for more efficient and innovative ways of conducting clinical trials.

### 6.3. Master Protocols and Adaptive Trial Designs

Platform, basket and umbrella trials are typical formats for a “master protocol”. Initially designed and used in oncology trials, this design enabled the simultaneous evaluation of multiple therapeutic drugs across multiple biomarker-defined populations in a single clinical trial infrastructure. This approach is widely reviewed in the literature [69,70,71,72] and will not be discussed in detail here. However, this strategy is now being employed in therapeutic areas such as chronic pain management [73], autoimmune dysfunctions [74] and COVID-19 [75], to name a few.

Bayesian adaptive trial design is used in evaluating personalized therapies in a range of diseases [76,77,78,79]. This design uses an adaptive approach, which allows for real time outcomes to influence ongoing treatment assignment probabilities, contributing to enhanced flexibility and efficiency. By defining early stopping rules, ineffective treatments are also terminated at an earlier point in time, such that more patients can be subsequently treated with effective treatments. In adaptive trials, biomarkers are built into the protocol, to be used in guiding patient treatments.

### 6.4. Evaluating Biomarkers in Personalized Therapies

Even after a CDx is approved and launched, delivering the treatment to patients in the real-world clinic still presents as a hurdle [80]. There is a growing need to evaluate and demonstrate the clinical utility of using biomarkers to guide treatment regimes in the clinic.

Many “precision medicine trials” in oncology have already been conducted or are ongoing [81]. These trials molecularly profile patients’ tumor using methods such as NGS, polymerase chain reaction, mass spectrometry and immunohistochemistry to evaluate genomic, transcriptomic, or proteomic makeup. Based on actionable molecular alterations or features, a molecular tumor board, or “expert panel” recommends a treatment regimen, or personalized therapy for patients. A prospective study conducted at two cancer centers in the US—I-PREDICT—evaluated such a protocol on patients with refractory malignancies [82]. A “matching score”, that evaluates the number of clinically actionable genetic alterations with a matched therapy available for a patient is commonly used in such trials to correlate with improvements in the disease control rate and survival parameters. The I-PREDICT study described that in heterogenous cancers harboring multiple genetic aberrations that have “matched therapies”, the administration of a combination of the matched therapies resulted in better patient outcomes.

In Japan, the National Cancer Center had also initiated a personalized medicine trial [83]. The primary objective of this prospective study was to evaluate the clinical utility of performing a CGP panel at the time of initial diagnosis of patients with solid tumors, as compared to the current standard of performing a CGP test only after completion of current standard of care.

A traditional single pharmaceutical-sponsored drug trial is relevant in establishing the safety and efficacy of new targeted therapies. However, demonstrating that the biomarker-based personalized approach confers more clinical benefit to patients over the standard of care, although a key bridging step, is still a budding field in many regions.

Even while the science may support the motive to shift toward a personalized approach, from the patient and government perspectives, assessing the value and cost effectiveness of the approach is yet another complex issue. An overall study of the cost effectiveness of precision medicine concluded that precision medicine interventions were more effective than existing standard of care, but this study also highlighted the lack of clinical data to support conclusions [84]. A specific study by Safonov et al. [85] developed and used an analysis tool to evaluate cost-utility of predictive biomarkers in oncology. They concluded that the value of a biomarker is driven primarily by clinical efficacy of the treatment and treatment cost. Cost-effectiveness studies in specific disease settings may provide the needed insight into the value of personalized therapies [86,87,88,89]. Cost effectiveness and value of personalized approaches involving more complex and innovative biotechnologies are also still yet to be studied in-depth. As newer approaches are available and clinical utility validated, the ability to evaluate personalized approaches with respect to standard of care will be important in translating the method from a hypothetical to a practical one.

Evolving complex trial designs mean the involvement of and collaboration between multiple different stakeholders. On top of just genomic data, multiple modes of biomarker information should ideally be comprehensively interpreted to diagnose a patient based on their *disease blueprint*. Patient information may not only exist from clinical trials but also possibly from health care databases. In recent years, wearable devices have also made it possible to collect sequential, ambulatory data. For personalized approaches to be efficiently delivered to patients, the industry will have to transform the way that clinical efficacy is validated against a background of multimodal biomarker data.

## 7. Proposals

The benefits and challenges of biomarker-driven or personalized approaches are apparent. In this review, we emphasized the importance of our proposed PTE framework. In Japan, however, there are still gaps to be filled. In the survey that our working group had conducted in 2020, one of the top challenges identified (via analysis of free text comments) was the uncertainty of the actual uptake of biomarker testing in the clinic (i.e., reforming the standard of care). We inferred that this top challenge may be intertwined with other challenges identified, such as high R&D costs involved in biomarker validation and development [11]. Based on the reviewed content and insights obtained from the survey, we make the following proposals.

### 7.1. RWD Usage

Since 2017, the Pharmaceuticals and Medical Devices Agency (PMDA) has issued several guidance and points-to-consider documents relating to the use of real world data and evidence in a regulatory setting [90], however no specific information or use-cases were available on biomarker translation or qualification. Based on our survey, there are two major areas in which improvement is needed in RWD usage:

(1) Access and (2) mining and utilization.

Access—As databases such as disease registries are established in Japan [17,91,92], more biomarker, multi-omics data as well as electronic health data will accumulate. The content of these databases should be poised to be shared openly and efficiently, such that the curated data can be leveraged to its maximum.

Mining and Utilization—Data-driven and computational approaches provide deep insight into disease pathophysiology and this insight is a factor of success in drug development. Despite this, the usage of RWE in clinical development is still in its immature phases in Japan. AI and machine learning is being applied to biomedical data for integration and modeling [93]. However, such capabilities may not typically be available within traditional pharmaceutical organizations. The establishment of platforms for the effective mining of biomedical big data will require significant scientific and technical expertise in new fields. We emphasize that there are organizations taking the lead in this direction and have reported positive results [14,15].

Latest computational approaches and algorithms as well as results should be shared as openly as possible. Integration of healthcare data with quality control and analysis processes could be also achieved by government-led efforts for increased standardization and uniformity.

### 7.2. Regulations and Infrastructure

As the types and scope of biomarker use cases increase, there is a constant need for new guidance and policy to guide the industry in the development process. For example, points to consider when establishing and validating quantitative or semi-quantitative biomarker cut points is a topic still requiring expert consensus. National regulatory bodies should consider regularly updating policies and guidance coordinated with the latest biomarker use cases, and in harmonization with efforts from other global agencies. There are currently some gaps for instance, in the requirements of the CDx submission and approval pathways among the health authorities in the US, Europe and Japan [94].

As pointed out earlier, studies that demonstrate value in biomarker-focused or personalized approaches over current standard of care, are lacking. In such cases, it is especially important for stakeholders including key opinion leaders, government agency, and regulatory bodies to be aligned on the current unmet medical needs and agree on how biomarker-driven approaches can (or cannot) help address these needs. A framework for defining and evaluating “clinical benefit”, should be established, perhaps for each disease setting. This will instill more clarity and assurance on the direction that the industry should take in R&D efforts.

### 7.3. Evolution of the “Clinical Trial”

**Figure 2:**
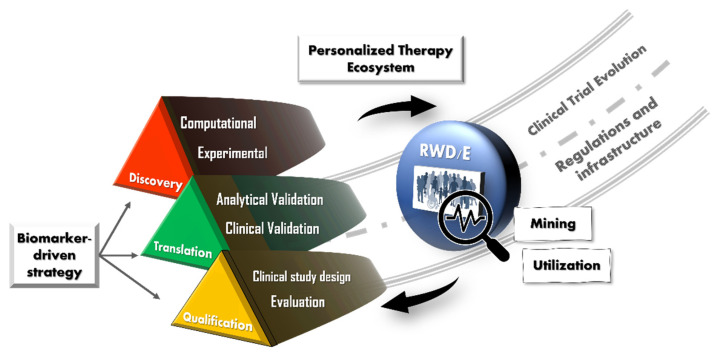
Roadmap for biomarker-driven drug development for personalized therapies.

Prospective platform and innovative trial designs described in the Qualification section above will be crucial in evaluating the utility of a true *disease blueprint*-based diagnosis in the clinic. In this climate, as we move away from the “one-drug-fits-all” and turn to the “personalized approach”, the “global clinical trial” becomes lacking and counterintuitive. While pharma-sponsored global clinical trials remain important in bringing new therapeutic agents to market, new-age personalized medicine trial designs should aim to fulfil the local regulatory, infrastructural, and medical needs. The industry therefore needs to re-evaluate collaboration frameworks, each stakeholder in drug development and healthcare having to pivot in terms of strategy to meet this need. We summarize this concept in Figure 2.

## 8. Discussion and Conclusions

As described thus far, reliable clinical biomarkers are at the core of personalized therapy. There is accumulating scientific evidence now especially in oncology that using clinically validated biomarkers to select patients for targeted therapies in precision medicine trials result in better outcomes. Our working group also performed an in-depth review on the medical needs for personalized approaches in non-oncology diseases [95].

While there is inertia to making significant investments in new technology and capabilities, there is evidence that high R&D investments reap high R&D output [96]. Simultaneously, to ensure that industry R&D investments can have the potential to be effectively translated into improved healthcare practices, all stakeholders need to collaborate in new and innovative ways to embrace change.

In three independent reviews by three pharmaceutical organizations, the authors reported that leveraging some form of genomic data or experimental data-driven approach in identifying the appropriate drug targets are an important part of organizational drug development strategies [13,14,15]. An industry-wide review conducted by Shih et al. [97] also corroborated the conclusion that therapies targeting well-validated biological mechanisms were a common feature of successful projects. Additionally, in two of the reviews, the use of validated biomarkers as intermediary outcome readouts was also part of the success strategy. Projects with a patient selection strategy had a higher chance of success than those without one [14]. Another common feature of the reported strategies is the “shift” that organizations are making to focus on understanding disease heterogeneity to select higher quality drug targets and refine patient selection strategies.

Although anecdotal, these examples support our concept of the importance of data-driven approaches in refining biomarker-driven drug development.

Within the restraints of current industry practices and frameworks, it may be that drug makers cannot “see the light at the end of the tunnel” and this is creating barriers to investment decisions, especially in smaller organizations that have limited resources.

In 2010, a Biomarker Task Force comprising multi-disciplinary representatives from the NCI and FDA, published a recommendation paper for incorporating biomarkers in early phase clinical trials with the goal of identifying exciting and novel agents [98]. The recommendations to sponsors revolved around the need for incorporating well thought-out and prioritized biomarker plans in clinical development programs. Institutional investigators were seen as a valuable collaborative resource for biomarker testing capabilities. Eleven years on, the drug development industry has effectively brought the biomarker into the clinic to provide personalized medicines to patients. Drug development programs now also typically have an accompanying biomarker strategy. Biomarkers being validated and tested in central reference laboratories are now a part-and-parcel of drug development programs. Banking of clinical samples collected during clinical trials are a routine procedure, and collaborations between sponsor and academia for exploratory biomarker research are a norm.

The recommendation from 2010 to prioritize biomarkers based on “solid science and the needs of patients” resounds with the central concept of our paper. In today’s context we have more robust methods to achieve this by tapping on to the mass of biomedical big data that is accumulating.

The personalized therapy approach is expected to increase the quality of diagnoses and reduce or prevent the prescription of ineffective treatments. This could ultimately translate to reductions in mortality rates, hospitalization periods and overall societal burden. The use of RWD to make decisions in an evidence-based manner will also help channel valuable resources into scientifically sound research causes. We encourage that all involved in healthcare and drug development be conscious of the PTE—a framework that links patients, clinicians, industry, academia, and government—and collaborate to bring healthcare to the next level.

## Figures and Tables

**Table 1:** Summary of approaches taken in biomarker and drug target discovery.

Approach	Specific Method	Source of Data/Samples	Applications in Drug Development
Computational	Genome-wide association studies (GWAS) Quantitative systems pharmacology (QSP) Network modeling	Large-scale omics data initiatives (e.g., 100,000 genomes project, ToMMo, etc.) Curated omics databases (e.g., TCGA, JGCA, SCRUM-Japan, etc.)	Insights into biology/disease pathology New target identification Identify disease-associated biomarkers Drug repurposing
Experimental	Patient-derived xenograft models Patient-derived iPSC models	Biobanks Clinical trial banked samples	New target screening/identification/optimization In vitro disease modeling
